# Human mesenchymal stromal cells do not express ACE2 and TMPRSS2 and are not permissive to SARS‐CoV‐2 infection

**DOI:** 10.1002/sctm.20-0385

**Published:** 2021-01-26

**Authors:** Maria A. Avanzini, Manuela Mura, Elena Percivalle, Francesca Bastaroli, Stefania Croce, Chiara Valsecchi, Elisa Lenta, Giulia Nykjaer, Irene Cassaniti, Jessica Bagnarino, Fausto Baldanti, Marco Zecca, Patrizia Comoli, Massimiliano Gnecchi

**Affiliations:** ^1^ Cell Factory Fondazione IRCCS Policlinico San Matteo Pavia Italy; ^2^ Pediatric Hematology Oncology Fondazione IRCCS Policlinico San Matteo Pavia Italy; ^3^ Intensive Cardiac Care Unit and Laboratory of Experimental Cardiology for Cell and Molecular Therapy Fondazione IRCCS Policlinico San Matteo Pavia Italy; ^4^ Molecular Virology Unit, Microbiology and Virology Department Fondazione IRCCS Policlinico San Matteo Pavia Italy; ^5^ Department of Molecular Medicine Unit of Cardiology, University of Pavia Pavia Italy; ^6^ General Surgery I Fondazione IRCCS Policlinico San Matteo Pavia Italy; ^7^ Department of Medicine University of Cape Town Cape Town South Africa

**Keywords:** adult stem cells, angiotensin, cellular therapy, fetal stem cells, mesenchymal stromal cells (MSCs)

## Abstract

Anti‐inflammatory and immune‐modulatory therapies have been proposed for the treatment of COVID‐19 and its most serious complications. Among others, the use of mesenchymal stromal cells (MSCs) is under investigation given their well‐documented anti‐inflammatory and immunomodulatory properties. However, some critical issues regarding the possibility that MSCs could be infected by the virus have been raised. Angiotensin‐converting enzyme 2 (ACE2) and type II transmembrane serine protease (TMPRSS2) are the main host cell factors for the severe acute respiratory syndrome‐coronavirus 2 (SARS‐CoV‐2), entry, but so far it is unclear if human MSCs do or do not express these two proteins. To elucidate these important aspects, we evaluated if human MSCs from both fetal and adult tissues constitutively express ACE2 and TMPRSS2 and, most importantly, if they can be infected by SARS‐CoV‐2. We evaluated human MSCs derived from amnios, cord blood, cord tissue, adipose tissue, and bone marrow. ACE2 and TMPRSS2 were expressed by the SARS‐CoV‐2‐permissive human pulmonary Calu‐3 cell line but not by all the MSCs tested. MSCs were then exposed to SARS‐CoV‐2 wild strain without evidence of cytopathic effect. Moreover, we also excluded that the MSCs could be infected without showing lytic effects since their conditioned medium after SARS‐CoV‐2 exposure did not contain viral particles. Our data, demonstrating that MSCs derived from different human tissues are not permissive to SARS‐CoV‐2 infection, support the safety of MSCs as potential therapy for COVID‐19.

## Graphical abstract

SARS‐CoV‐2 enters into host cells through ACE2 and TMPRSS2. Mesenchymal stromal cells (MSCs) derived from amnios (A), cord blood (CB), cord tissue (CT), adipose tissue (AT) and bone marrow (BM) do not express these proteins, as demonstrated by RT‐qPCR, western blot, ELISA and immunofuorescence. They are not infected by either spike pseudovirus, or by SARS‐CoV‐2 wild strain.

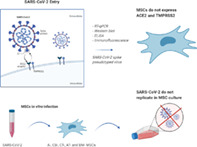

**Significance statement**

Human mesenchymal stromal cells (hMSCs) are currently under investigation for the treatment of COVID‐19. However, the potential safety profile of hMSCs in this context has never been defined since none has described if they express ACE2 and TMPRSS2, the main host cell factors for SARS‐CoV‐2 entry, and if they can be infected by SARS‐CoV‐2. This study provides the first evidence that ACE2 and TMPRSS2 are not expressed in hMSCs derived from both adult and fetal human tissues and, most importantly, that hMSCs are not permissive to SARS‐CoV‐2 infection. These results support the safety of MSCs as potential therapy for COVID‐19.

## 1 INTRODUCTION

In December 2019, an outbreak caused by a novel coronavirus, later named SARS‐CoV‐2, occurred in China and rapidly spread throughout several other countries, becoming pandemic.^1^ COVID‐19, the disease caused by SARS‐CoV‐2, mainly affects the respiratory system and can progress to respiratory distress syndrome (ARDS), a fatal condition in more than 50% of the cases.^1^ Up to 30% of COVID‐19 patients may develop cardiac damage due to acute coronary syndrome, septic heart, or acute myocarditis.^2^, ^3^, ^4^ As there are no specific therapeutics for treating COVID‐19, in particular for the most severe cases complicated by ARDS or acute fulminant myocarditis, new innovative therapeutic approaches are urgently needed. Anti‐inflammatory drugs have been proposed as possible approaches and it has been suggested that immunosuppressive therapy may mitigate the manifestations of COVID‐19.^5^, ^6^ Mesenchymal stem cells (MSCs) possess immunomodulatory properties as demonstrated by numerous in vitro and animal model studies. These effects are mediated by cytokines and soluble factors able to modulate the systemic but also the tissue inflammatory response.^7^ In particular, when administered intravenously, most MSCs lodge in the pulmonary vascular bed where they survive for at least a few days.^8^ Importantly, several clinical studies, including phase III trials, documented their efficacy in the control of graft‐vs‐host disease in recipients of allogeneic hematopoietic stem cell transplantation, and there are evidences also in other immune‐mediated disorders.^7^ In addition, there are existing preclinical and a few preliminary feasibility and safety clinical studies supporting further investigation of cell‐based therapies, particularly with MSC, or the MSC‐derived secretome, for potential treatment of ARDS and acute myocarditis.^7^, ^8^, ^9^ For all these reasons, it has been hypothesized that the administration of MSCs may be useful in the treatment of severe cases of COVID‐19. A pilot study conducted in China on seven patients affected by SARS‐CoV‐2 pneumonia has reported feasibility and safety of MSC therapy,^10^ and a total of 39 phase I/II trials testing MSC‐therapy for COVID‐19 are currently registered in ClinicalTrials.gov. However, infusion of MSCs in the presence of active viraemia has raised some critical issues regarding the possibility that the virus may infect MSCs, causing not only lack of efficacy but also possible deleterious effects.

Angiotensin‐converting enzyme 2 (ACE2) is the main host cell receptor for SARS‐CoV‐2 entry, and the virus uses the host cell transmembrane serine protease II (TMPRSS2) for Spike envelope protein priming.^11^ It is known that ACE2 and TMPRSS2 are present on the surface of several human cells, such as alveolar cells and capillary endothelium, while immune cells, such as T and B lymphocytes, and macrophage are negative for ACE2.^12^ Whether human MSCs of any origin constitutively express ACE2 and/or TMPRSS2 is so far unclear. Leng et al^10^ claimed the absence of ACE2 and TMPRSS2 expression on the umbilical cord‐derived MSC infused in their study, even though a clear demonstration was not reported. In addition, SARS‐CoV‐2 could also use other, possibly unknown, receptors for cellular entry.

Accordingly, the aim of the present work was to evaluate whether human MSCs from various sources express ACE2 and TMPRSS2 and, most importantly, if they are permissive to SARS‐CoV‐2 infection.

## 2 MATERIALS AND METHODS

An expanded method section is available as Supporting Information.

### 2.1 Cell culture

The MSC lines were previously isolated from amniotic membrane of human placenta, cord blood, cord tissue, bone marrow, and adipose tissue, expanded and characterized.^13^, ^14^, ^15^, ^16^, ^17^ All the MSCs fulfill the criteria set by the International Society for Cell & Molecular Therapy (ISCT).^18^ To perform the experiments, we used passage 3 to 5 MSCs. Conditioned media were generated as described^13^, ^19^ with some minor modification described in the Supporting Information Methods, in order to evaluate the soluble amount of ACE2. The human lung Calu‐3 (ATCC HTB‐52) and the African green monkey kidney VERO E6 (VERO C1008; ATCC CRL‐1586) cell lines were purchased and maintained as indicated by ATCC (www.lgcstandards-atcc.org).

### 2.2 SARS‐CoV‐2 spike pseudotyped retrovirus production and MSC infection

SARS‐CoV‐2 spike pseudotyped retroviral particles were produced by cotransfection of 293T cells (ThermoFisher) with these following three plasmids: a replication‐deficient retroviral vector FCQ pMM2‐eGFP expressing the green fluorescent protein (GFP)^20^; a packaging vector pUMVC (#8449 Addgene), and an envelope vector 2019‐nCoV Spike ORF mammalian expression plasmid (VG40589‐UT Sino Biologicals), or pCMV‐VSV‐G (#8454 Addgene) expressing the vesicular stomatitis virus glycoprotein (VSV‐G) as a positive control. MSCs were incubated with Spike or VSV‐G pseudotyped viral particles for 24 hours.

### 2.3 Infection with SARS‐CoV‐2 wild strain

MSCs, Calu‐3, and VERO E6 were infected with 100 μL (100 TCID_50_/mL) of a previously titrated SARS‐CoV‐2 wild strain, isolated from an infected patient. The virus was incubated for 1 hour and then removed; the medium was changed every 3 days. Cells were scored every other day and for 1 week using a light microscope to detect the appearance of cell rounding, detachment, degeneration, and/or syncytium formation, called hereafter cytopathic effect (CPE). To verify if the cells tested can be infected and allow SARS‐CoV‐2 replication even in the absence of CPE, at day 7 from infection, supernatants from each MSC, Calu‐3, and VERO E6 culture were collected and inoculated into VERO E6. CPE occurrence was monitored for 1 week.

### 2.4 Statistical analysis

All results are reported as mean ± SD and the data were analyzed with a one‐way or two‐way analysis of variance followed by Bonferroni all pair‐wise multiple comparison test using the InStat software (GraphPad Software, Inc., San Diego, California; http://www.graphpad.com). *P* values less than .05 were considered statistically significant.

## 3 RESULTS

**FIGURE 1:**
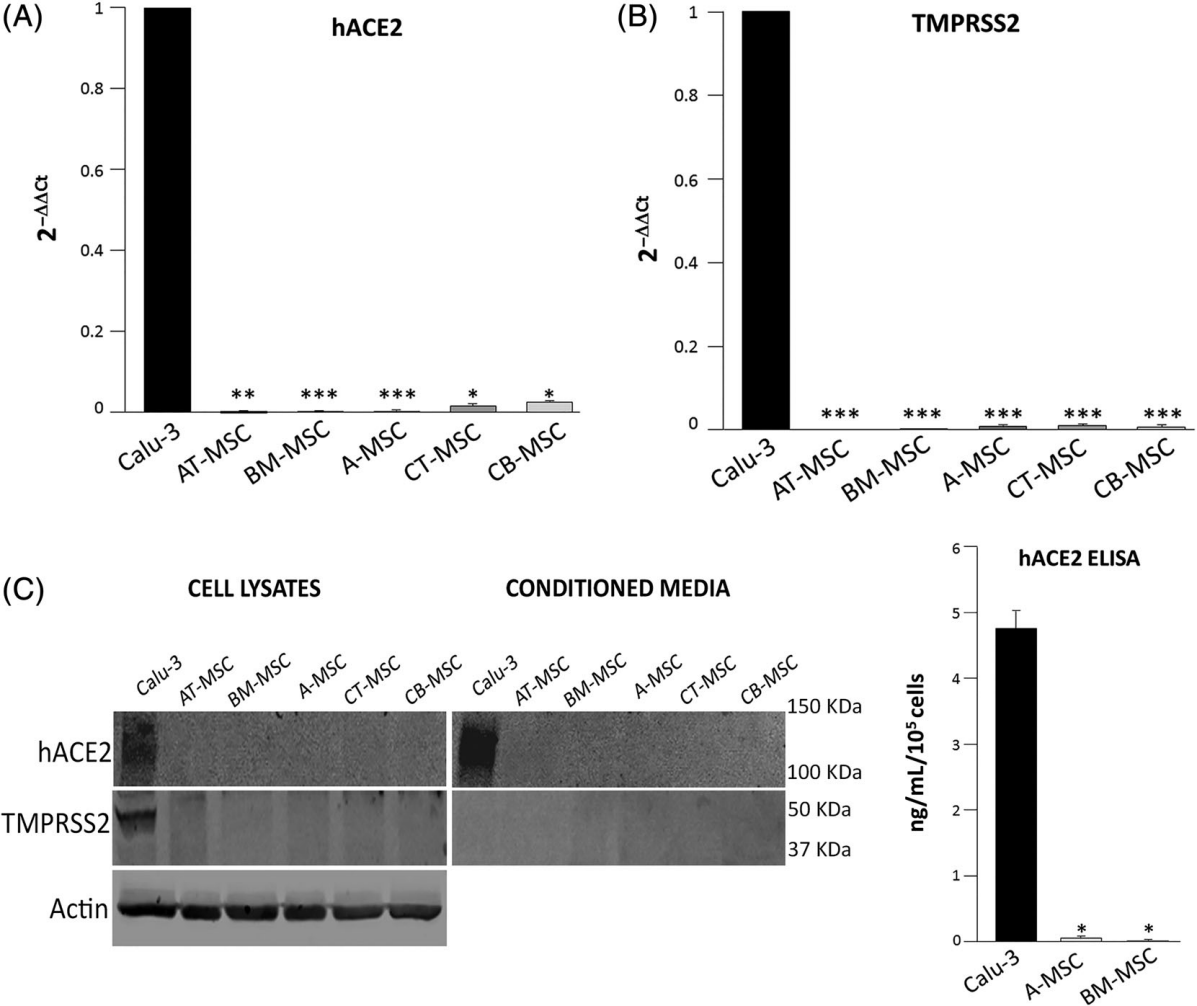
hACE2 and TMPRSS2 expression in human MSCs from fetal and adult tissues. A, Relative quantification by quantitative reverse transcription PCR (RT‐qPCR) and the 2^−ΔΔCt^ method of ACE2 (left) and TMPRSS2 (right) mRNA levels in MSCs from cord blood (CB‐MSC, n = 2 lines), cord tissue (CT‐MSC, n = 2 lines), amnios (A‐MSC, n = 4 lines), bone marrow (BM‐MSC, n = 4 lines), and adipose tissue (AT‐MSC, n = 2 RNA samples from the same line), compared with the Calu‐3 cell line. Each sample was loaded in triplicate. Columns represent mean 2^−ΔΔCt^ values and SD. The glyceraldehyde 3‐phosphate dehydrogenase (GAPDH) was used as a reference gene. *P < .05, **P < .01, ***P < .001 vs Calu‐3. B, Representative Western blot of hACE2 and TMPRSS2 in cell lysates (left) and serum free 48 hours‐conditioned media (right) of the different MSC types, and the positive control cell line Calu‐3. Actin was used as loading control for cell lysates. The amount of conditioned media loaded for each sample was obtained from 1 × 10^5^ cells. C, ACE2 protein quantification by enzyme‐linked immunoadsorbent assay (ELISA) in serum‐free 48 hours‐conditioned media generated by 1 × 10^5^ MSCs. The bars represent the mean and SD of triplicates obtained from a single line of Calu‐3 and 4 distinct lines of both A‐MSCs and BM‐MSCs. *P < .001 vs Calu‐3. MSCs, mesenchymal stromal cells

We assessed ACE2 and TMPRSS2 expression on human fetal MSCs derived from amniotic membrane of placenta (A, n = 4), cord blood (CB, n = 2) or cord tissue (CT, n = 2), and human adult MSCs derived from bone marrow (BM, n = 4) or adipose tissue (AT, n = 1). The lung epithelial cell line Calu‐3, which expresses high levels of both ACE2 and TMPRSS2 and is permissive to SARS‐CoV‐2 infection and nonlytic replication,^21^ was used as positive control. Compared with Calu‐3, the levels of ACE2 and TMPRSS2 mRNAs in all MSC lines considered were around 100‐fold and 200‐fold lower, respectively (Figure 1A). ACE2 and TMPRSS2 protein expression in MSC lysates was undetectable by Western blot (Figure 1B). Finally, we were unable to detect any soluble amount of ACE2 in MSC‐derived conditioned media by both Western blot and enzyme‐linked immunoadsorbent assay (ELISA) (Figure 1B,C).

**FIGURE 2:**
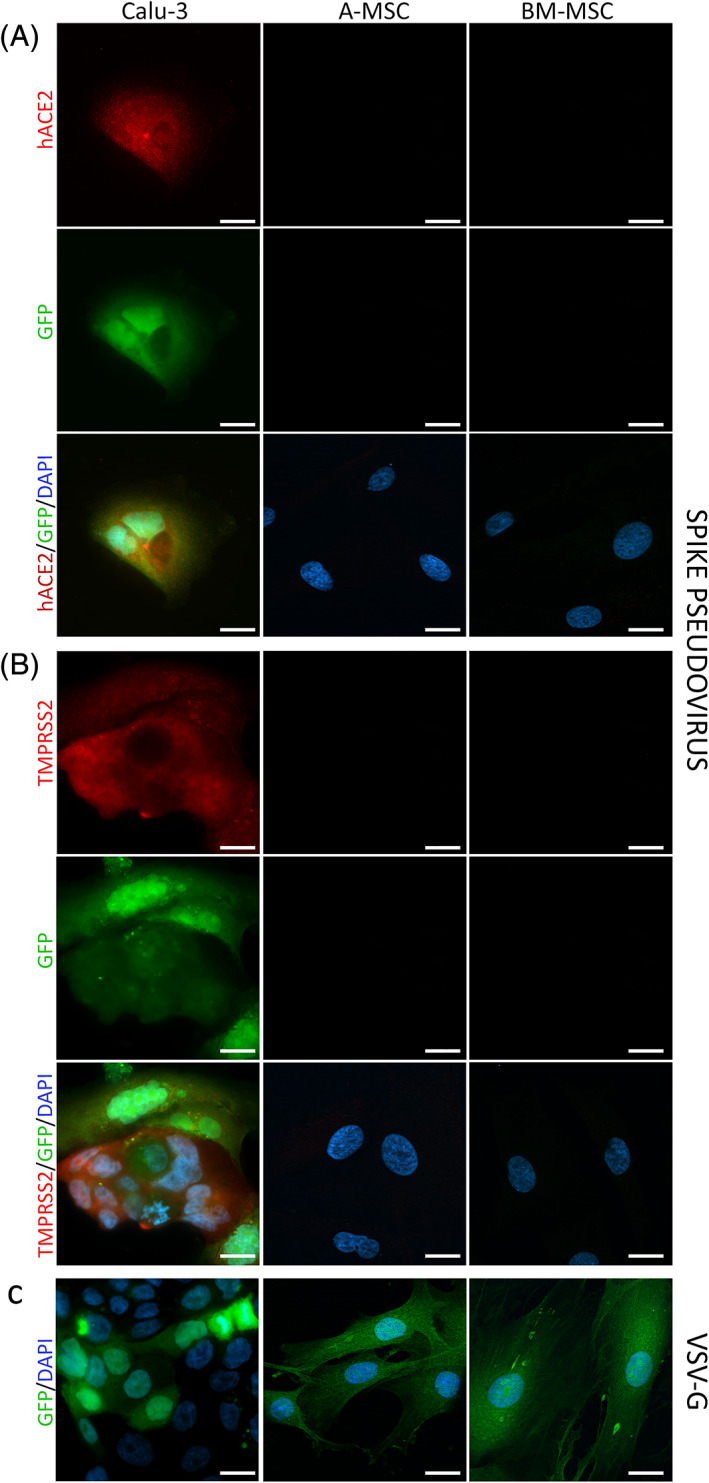
MSC infection with a SARS‐CoV‐2 Spike pseudovirus. A‐MSCs, BM‐MSCs, and Calu‐3 were transduced with a replication‐defective, green fluorescent protein (GFP)‐tagged, pseudotyped retrovirus bearing the SARS‐CoV‐2 spike envelope protein (Spike pseudovirus; A, B), or the pantropic vesicular stomatitis virus glicoprotein (VSV‐G; C). The green signal is present if cells are infected. Costaining for ACE2 (A) or TMPRSS2 (B) is shown in red. Nuclei were counterstained with the nuclear dye 4′,6‐diamidino‐2‐phenylindole (DAPI Blue). Scale bar = 20 μm

To substantiate the absence of ACE2 and TMPRSS2 in fetal and adult MSCs, we transduced both A‐MSCs and BM‐MSCs, as representative cell populations, with a replication‐defective, GFP‐tagged, pseudotyped retrovirus bearing the SARS‐CoV‐2 spike envelope protein. This pseudovirus does not go through a lytic replication and does not induce CPE, but it shares the same host cell factors for viral entry with the authentic SARS‐CoV‐2. As expected, Calu‐3 were infected by the spike‐pseudotyped virus and turned green, confirming that the assay properly worked (Figure 2). On the contrary, neither A‐MSCs nor BM‐MSCs were infected by the pseudovirus (Figure 2). A‐MSCs, BM‐MSCs, and Calu‐3 were all susceptible to entry driven by the pantropic VSV‐G, confirming the specificity of the assay (Figure 2). Finally, immunofluorescent analysis confirmed the expression of ACE2 and TMPRSS2 only in the permissive cell line Calu‐3, whereas no expression was documented in fetal and adult human MSCs (Figure 2).

**FIGURE 3:**
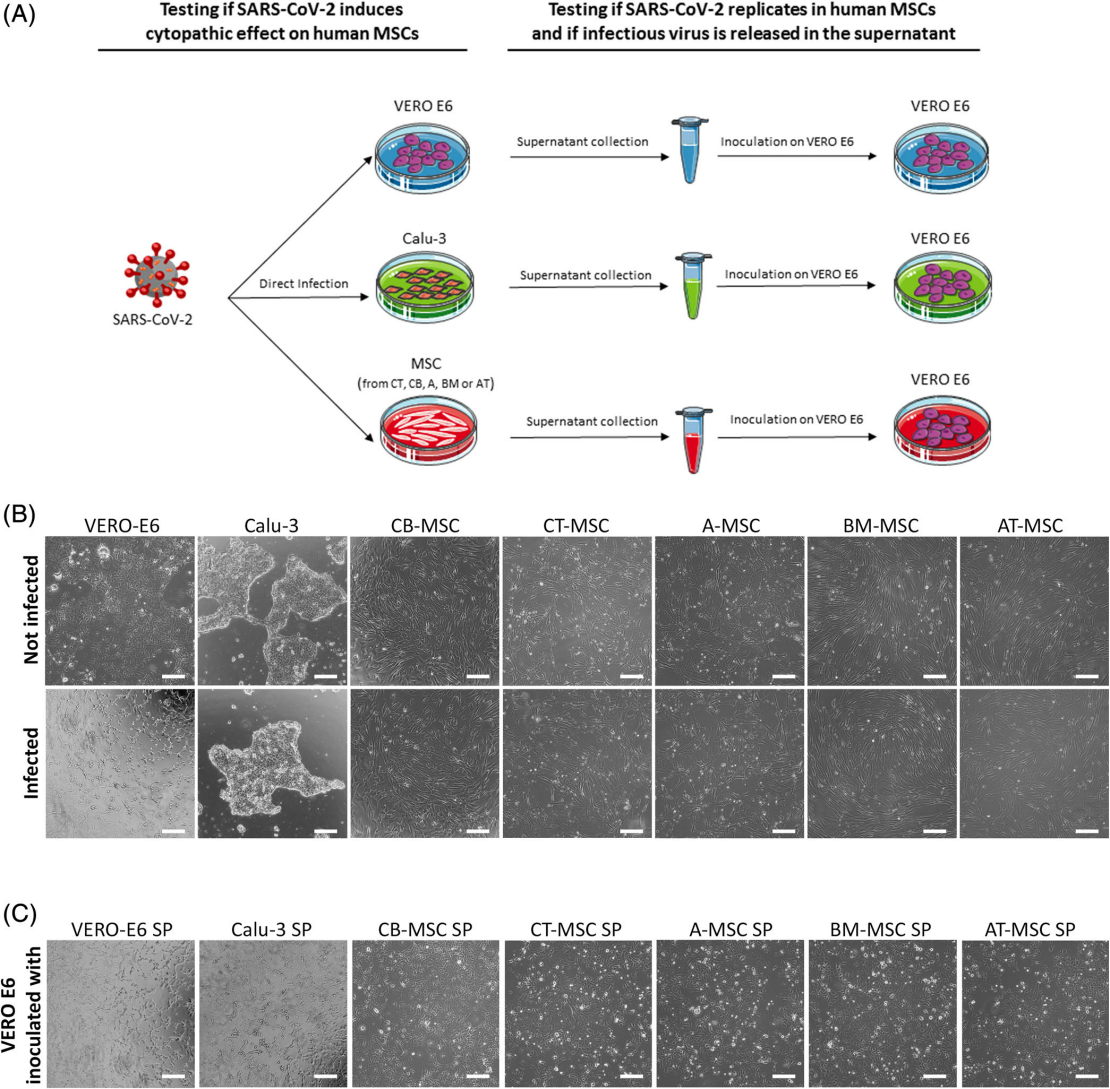
MSC infection with SARS‐CoV‐2. A, Experimental design. First, MSCs from CB, CT, A, BM and AT, and the permissive cell lines VERO E6 and Calu‐3 were incubated with SARS‐CoV‐2 wild‐type strain. CPE defined as cell rounding, detachment, degeneration, and/or syncytium formation was assessed by microscopic analysis, and the cells maintained in culture for 7 days, changing the medium every three 3 days. Then, supernatants were collected and tested in a reinoculation experiment on VERO E6. B, Representative phase contrast images of VERO‐E6, Calu‐3, and hMSCs before (not infected—upper panels) and after exposure to SARS‐CoV‐2 (infected—lower panels). Scale bar = 200 μm. hMSCs and Calu‐3 showed no signs of CPE and maintained their typical spindle‐shaped and epithelial morphology, respectively, whereas VERO E6 displayed clear signs of CPE, since almost all the cells were round or fused into syncytia or detached. C, Representative phase contrast images of VERO E6 cells after exposure to supernatant generated by the infected cell lines respectively indicated on top of each frame. Scale bar = 200 μm. CPE, cytopathic effect; hMSCs, human mesenchymal stromal cells; MSCs, mesenchymal stromal cells

To exclude that SARS‐CoV‐2 can infect MSCs through other host factors/receptors, we infected fetal and adult human MSCs of different origin with a SARS‐CoV‐2 wild strain under two different conditions: as adherent monolayer or cellular suspension (Figure 3A). All MSCs infected in adhesion showed the typical spindle shape morphology with no signs of CPE (Figure 3B and Table 1). Also, MSCs infected in suspension and seeded in 24 well plates were found adherent to plastic and showed no CPE starting from the day postinoculum up to 7 days (data not shown). Conversely, a 100% CPE was detected in the control VERO E6 cell line (Figure 3B and Table 1). As expected,^21^ we did not observe CPE in Calu‐3 cells for the entire observation period (Figure 3B and Table 1). At 7 days after infection, supernatants from all experiments were collected and tested in a reinoculation experiment in VERO E6 cells (Figure 3A). As expected, a typical CPE was evident in all the wells inoculated with supernatants collected from infected VERO‐E6 and Calu‐3 cultures (Figure 3C and Table 1). On the contrary, none of the supernatant collected from the different MSC cell lines induced CPE, demonstrating the absence of viral replication inside the MSC lines and, consequently, the absence of SARS‐CoV‐2 infection (Figure 3C and Table 1).

**TABLE 1:** Cytopathic effect in human MSCs, Calu‐3, and VERO E6 cell lines

Cell line	n	CPE after 1 wk of SARS‐CoV‐2 exposure			CPE detected in VERO E6 inoculated with supernatants		
		1 × 10^5^	5 × 10^4^	2.5 × 10^4^	1 × 10^5^	5 × 10^4^	2.5 × 10^4^
CB‐MSC	2	N	N	N	N	N	N
CT‐MSC	2	N	N	N	N	N	N
A‐MSC	1	N	N	N	N	N	N
AT‐MSC	1	N	N	N	N	N	N
BM‐MSC	4	N	N	N	N	N	N
Calu‐3	2	N	N	N	P	P	P
VERO E6	2	P	P	P	P	P	P

*Note*: The presence or absence of virus‐induced CPE is reported in the table. Cytopathic effect was defined as cell rounding, detachment, degeneration, and/or syncytium formation after 1 week of direct virus infection or after inoculation with supernatant collected from previously infected cells.

Abbreviations: CPE, cytopathic effect; MSCs, mesenchymal stromal cells; N, negative CPE; P, positive CPE.

## 4 DISCUSSION

The recent emergence of the COVID‐19 pandemic and the absence of specific and validated therapeutic agents against this disease, prompted the search for new therapies able to hamper the strong immune reaction and the life‐threatening complications occurring in the most severe cases. The inflammatory nature of COVID‐19 points toward a solid rationale for the use of MSCs. Indeed, current understanding of MSC mechanisms of action is that most of their beneficial effects in repair from injury occur through secretion of cytoprotective, repair‐promoting, and immunoregulatory factors.^22^ Some examples include the induction of M2 macrophages, inhibition of natural killer cell proliferation and their cytotoxic function, anti‐inflammatory cytokine production, and promotion of T regulatory cell generation.^8^ Overall, these immunomodulatory effects can facilitate the resolution of inflammatory processes, including those characterizing ARDS and acute myocarditis. Furthermore, MSCs have constitutively low immunogenicity allowing off‐the‐shelf allogeneic use and there is a strong track record of safety for use in a range of diseases. In particular, MSCs from different sources including bone marrow, adipose, cord blood, and placental tissues, have shown promising results in experimental models of lung diseases, following either systemic or direct endobronchial administration.^8^ Phase I and II trials have demonstrated feasibility and safety of systemic administration of MSCs in non‐COVID ARDS patients and inflammatory cardiomyopathy.^8^, ^9^ As alveolar epithelium and capillary endothelium are major sites of viral replication during COVID‐19 disease, it was hypothesized that MSCs retained within the lungs and the capillaries, for instance after intravenous infusion, may rapidly undergo infection and relative virus‐mediated lysis, with significant decrease in therapeutic efficiency. For this reason, determining if human MSCs can be infected by SARS‐CoV‐2 is of crucial importance.

Stem cells are generally resistant to viral agents^23^ but infection of MSCs by avian influenza or herpesviruses has been reported,^24^, ^25^ and a similar concern has been raised for the SARS‐CoV‐2. So far, evaluation of MSC infectiveness by coronaviruses, in particular by SARS‐CoV‐2, has not yet been investigated and described. There is only one study in which absence of the viral host cell factors ACE2 and TMPRSS2 expression on human umbilical cord blood‐derived MSCs is claimed, but this cannot be considered a surrogate indicating a condition of refractoriness to infection.^10^ Indeed, the presence on MSCs of a different receptor able to mediate viral entry cannot be ruled out. Moreover, if all human MSC types express or not ACE2 is still a matter of debate and solid data are missing.

Using a variety of assays, we unequivocally demonstrated that human MSCs do express neither ACE2 nor TMPRSS2. Most importantly, using a direct infection assay, our study provides the first evidence that human MSCs derived from fetal and adult tissues are not permissive to SARS‐CoV‐2 infection.

## 5 CONCLUSION

Our data demonstrating that MSCs derived from different human tissues are resistant to SARS‐CoV‐2 infection are important to support the use of MSCs as a possible useful tool to down‐modulate the immune hyper‐activation in COVID‐19 patients, and to contrast the pro‐fibrotic mechanisms that lead to the severe long‐term pulmonary sequelae increasingly observed in patients recovering from acute infection.

## CONFLICT OF INTEREST

F.B. declared consultant/advisory role with Humabs, Biotest, Shire, DiaSorin, MSD Qiagen; research funding from AB Analitica, NTP, Qiagen, Elitechgroup, and DiaSorin. The other authors declared no potential conflicts of interest.

## AUTHOR CONTRIBUTIONS

M.A.A.: conception and design, collection and assembly of data, data analysis and interpretation, manuscript writing, financial support, final approval of manuscript; M.M., E.P.: conception and design, collection and assembly of data, data analysis and interpretation, manuscript writing; F. Bastaroli, S.C., C.V., E.L., G.N.: collection and assembly of data, data analysis and interpretation; I.C., J.B., F. Baldanti, M.Z., P.C.: financial support and final approval of manuscript; M.G.: conception and design, financial support, data analysis and interpretation, manuscript writing, final approval of manuscript.

## Supporting information


**Appendix** **S1**. Supporting Information

## Data Availability

The data that support the findings of this study are available on request from the corresponding author.
