# Supplementary material for: Human mesenchymal stromal cells do not express ACE2 and TMPRSS2 and are not permissive to SARS‐CoV‐2 infection
Source: Stem Cells Transl Med. 2021 Jan 26;10(4):636–42. doi: 10.1002/sctm.20-0385 (PMC7753681; doi:10.1002/sctm.20-0385)
Supplement: Supplementary file 1 — Appendix S1. Supporting Information [file SCT3-10-636-s001.docx]

**SUPPLEMENTAL MATERIAL**

**Human Mesenchymal Stromal Cells do not express ACE2 and TMPRSS2 and are not permissive to SARS-CoV-2 infection**

* Maria A. Avanzini, *Manuela Mura, *Elena Percivalle, Francesca Bastaroli, Stefania Croce, Chiara Valsecchi, Elisa Lenta, Giulia Nykjaer, Irene Cassaniti, Jessica Bagnarino, Fausto Baldanti, Marco Zecca, Patrizia Comoli, Massimiliano Gnecchi.

**MATERIALS AND METHODS**

**Cell culture**

All MSC types were cultured in alpha-minimum essential medium (α-MEM, Biowest) supplemented with 5% human platelet lysate (Cook Regentec), 2 mmol/l L-glutamine, 100 U/ml penicillin, and 100 mg/ml streptomycin (all from Gibco, ThermoFisher), 0,2% MycoZap PR (Lonza). Cells were detached at confluency using trypsin 0,05% in PBS 1X (ThermoFisher), and plated at the density of 2500 cells/cm^2^, or cryopreserved in Cryostor CS10 (BioLife Solutions).

The human lung Calu-3 (ATCC HTB-52) and the african green monkey kidney VERO E6 (VERO C1008; ATCC® CRL-1586™) cell lines were maintained in Eagle’s Minimum Essential Medium (EMEM, ATCC) supplemented with 10% fetal bovine serum (FBS – Sigma Aldrich), as indicated by ATCC ([www.lgcstandards-atcc.org](http://www.lgcstandards-atcc.org)). All cell lines were incubated at 37°C and 5% CO_2_ in a humidified atmosphere.

**Production of conditioned media**

Conditioned media from MSCs and Calu-3 were generated as follows: 90% confluent cells were fed with serum-free medium and incubated for 48 hours at 37°C. The medium was then collected, centrifuged at 4000 rpm to remove cell debris, and the cells were counted for normalization purposes. After that, the supernatant was transferred to dedicated ultrafiltration tubes with 3 KDa nominal molecular weight limit (Amicon Ultra-3K device; EMD Millipore, Bedford, MA) and 10-fold concentrated by centrifugation at 4000 rpm for 45 min at 4°C according to the manufacturer’s protocol.

**Reverse transcription, PCR and real Time qPCR**

Total RNA was extracted from MSCs and Calu-3 using PureZOL® Reagent (Biorad) according to the manufacturer's instructions and quantified with Nanodrop (Celbio). 500 ng of each RNA sample was reverse transcribed into cDNA with SuperScript IV Reverse Transcriptase (ThermoFisher). cDNAs were amplified by Real Time PCR with Power SYBR Green PCR Master Mix (Applied Biosystems, Carlsbad, California, USA) and the ABI Prism 7900HT Fast Real Time PCR System (Applied Biosystems). Data analysis was performed using the 2^-ΔΔCt^ relative quantification method, using the GAPDH as reference gene, and Calu-3 as reference sample. Primer pairs for human ACE2 (HP100185) and TMPRSS2 (HP101690) were purchased from Sino Biologicals. Primers for human GAPDH (Fw 5’-catgttccaatatgattccaccc-3’, Rev. 5’-gggatctcgctcctggaagat-3’) were designed by using the 'Primer3 input' software and the specificity of each primer was confirmed using the BLAST software (NCBI).

**Protein extraction, western blot, immunoblot**

Cells were lysed in ice-cold RIPA buffer supplemented with a Protease Inhibitor Cocktail (ThermoFisher), and the extracted proteins were quantified using a bicinchoninic acid method (BCA, Sigma Aldrich). Proteins from both cell lysates and conditioned media were then fractionated by SDS-PolyAcrylamide Gel Electrophoresis (SDS-PAGE), and hACE2 and TMPRSS2 were revealed by immunoblot. To perform the SDS-PAGE, 50 μg of protein samples or concentrated conditioned media from 1x10^5^ cells, were mixed with 12,5 μl of 4X Laemmli buffer (Bio-rad) and 2,5 μl of DTT 2M in a final volume of 50 μl, and heat-denatured at 95°C for 5 mins. Then they were fractionated on 7,5% or 12% Mini PROTEAN TGX Precast gels following manufacturer’s instructions (Bio-rad), and blotted onto a nitrocellulose membrane with the Transblot Turbo transfer system (Bio-rad).

For immunoblotting, membranes were blocked in Odyssey Blocking Buffer (Li-COR) diluted 1:1 in PBS 1X, and then incubated overnight at 4ºC with anti hACE2 antibody [SN0754] (GTX01160, Genetex), anti TMPRSS2 antibody [N2C3] (GTX100743, Genetex), diluted 1:1000, or anti actin [ACTN05 (C4)] antibody (ab3280, Abcam) diluted 1:400 in Odyssey Blocking Buffer 1:1 PBS 0,2%Tween 20. At the end of the incubation, membranes were washed three times for 5 mins in PBS 0.1% tween 20 and subsequently incubated at room temperature for 1 hour with anti rabbit IRDye800 or anti mouse IRDye680 -conjugated secondary antibodies (926-32211 or 926-32220, Li-COR) diluted 1:10000. After further three washes, bands were visualized with the Odyssey Infrared Imaging System (Li-COR).

**ELISA**

ACE2 levels in conditioned media generated by 1x10^5^ cells were quantified using the RayBio Human ACE-2 ELISA kit (ELH-ACE2, RayBiotech) following manufacturer’s instructions.

**Immunofluorescence**

Cells seeded on slides coated with gelatin 0,1% (Sigma Aldrich) were fixed in 4% paraformaldehyde for 12 mins, rinsed 2 times in PBS, and permeabilized in 0.1% Triton X-100 for 5 minutes. Samples were blocked with 1% bovine serum albumin (BSA) for 1 hour. The cells were incubated overnight at 4°C with anti hACE2 antibody [SN0754] (GTX01160, Genetex) or anti TMPRSS2 antibody [N2C3] (GTX100743, Genetex), both diluted 1:200 in 1% BSA, and then rinsed 3 times for 5 minutes with PBS. Further incubation was performed for 60 minutes at room temperature with goat anti rabbit Alexa 546 secondary antibody (Molecular Probes) diluted 1:500 in 1%BSA. The cells were rinsed three times more, mounted with ProLong Gold Antifade reagent with DAPI (Invitrogen), and analyzed with the AxioObserver.Z1 (Zeiss), and AxioVision REL.4.8 Software (Zeiss).

**SARS-CoV-2 spike pseudotyped retrovirus production and MSC infection**

SARS-CoV-2 spike pseudotyped lentiviral particles were produced in 293T cells (ThermoFisher). Briefly, 293T were split in 75 cm^2^ flasks at the seeding density of 4x10^6^ cells/flask, in DMEM high glucose (Biowest), supplemented with 10% FBS (Sigma Aldrich) 100 U/ml penicillin, and 100 mg/ml streptomycin (all from Gibco, ThermoFisher), 0,2% MycoZap CL (Lonza). The day after, they were co-transfected with 15 μg of each of the following plasmids, using the Calcium/Phosphate method: a replication-deficient retroviral vector FCQ pMM2-eGFP expressing the GFP (green fluorescent protein) (*Lee et al. 2020,* *doi: 10.1093/cvr/cvaa019*); a packaging vector pUMVC (#8449 Addgene), and an envelope vector 2019-nCoV Spike ORF mammalian expression plasmid (VG40589-UT Sino Biologicals), or pCMV-VSV-G (#8454 Addgene) espressing the vesicular stomatitis virus glycoprotein (VSV-G) as a positive control. Viral supernatants (10 ml for each flask) were harvested 72 hours after transfection and passed through 0.45 μm filters, to remove cell debris. 1ml of each virus was used to infect 2,5 x 10^4^ cells, seeded in one well of a 24-well plate.

**Infection with SARS-CoV-2 wild strain**

MSCs were seeded in 24 wells culture microplates (COSTAR, Corning Incorporated) at different concentrations (1x10^5^, 5x10^4^ and 2.5x10^4^) and incubated at 37°C, 5% CO_2_ in order to allow plastic adhesion. Subconfluent cells were infected with 100 µl (100 TCID _50_ /ml) of a previously titrated SARS-CoV-2 wild strain, isolated from an infected patient. The same number of cells in suspension were also infected with the same virus concentrations. The virus was incubated for 1 hour and then removed by changing the medium. Cells were scored every other day at a light microscope for one week to detect a cytopathic effect (CPE). During this week, the cell medium was changed every 3 days. As control, VERO E6 cell line was infected with the same amount of virus. We also evaluated Calu-3 in the same culture condition. After one week from infection, 200 µl of each supernatant from MSC, Calu-3 and VERO E6 culture were collected and inoculated into VERO E6 in a 24 well microplate. CPE appearance was monitored for another week.
